# Inner marginal strength of CAD/CAM materials is not affected by machining protocol

**DOI:** 10.1080/26415275.2021.1964969

**Published:** 2021-08-24

**Authors:** Julia Lubauer, Renan Belli, Fernanda H. Schünemann, Ragai E. Matta, Manfred Wichmann, Sandro Wartzack, Harald Völkl, Anselm Petschelt, Ulrich Lohbauer

**Affiliations:** a Friedrich-Alexander-Universität Erlangen-Nürnberg (FAU), Zahnklinik 1 – Zahnerhaltung und Parodontologie, Forschungslabor für dentale Biomaterialien, Erlangen, Germany; b Post-Graduate Program in Dentistry (PPGO), School of Dentistry, Federal University of Santa Catarina, Florianópolis, Brazil; c Friedrich-Alexander-Universität Erlangen-Nürnberg (FAU), Zahnklinik 2 – Prothetik, Labor für digitale Zahnmedizin, Erlangen, Germany; d Friedrich-Alexander-Universität Erlangen-Nürnberg (FAU), Lehrstuhl für Konstruktionstechnik, Erlangen, Germany

**Keywords:** CAD/CAM, strength, finite element

## Abstract

**Purpose:**

Here we aimed to compare two machining strategies regarding the marginal strength of CAD/CAM materials using a hoop-strength test in model sphero-cylindrical dental crowns, coupled with finite element analysis.

**Materials and Methods:**

Five CAD/CAM materials indicated for single posterior crowns were selected, including a lithium disilicate (IPS e.max^®^ CAD), a lithium (di)silicate (Suprinity^®^ PC), a polymer-infiltrated ceramic scaffold (Enamic^®^), and two indirect resin composites (Grandio^®^ Blocs and Lava™ Ultimate). A sphero-cylindrical model crown was built on CAD Software onto a geometrical abutment and machined using a Cerec MC XL system according to the two available protocols: *rough-fast* and *fine-slow*. Specimens were fractured using a novel hoop-strength test and analyzed using the finite element method to obtain the inner marginal strength. Data were evaluated using Weibull statistics.

**Results:**

Machining strategy did not affect the marginal strength of any restorative material tested here. Ceramic materials showed a higher density of chippings in the outer margin, but this did not reduce inner marginal strength. IPS e.max^®^ CAD showed the statistically highest marginal strength, and Enamic^®^ and Lava™ Ultimate were the lowest. Grandio^®^ Blocs showed higher performance than Suprinity^®^ PC.

**Conclusions:**

The *rough-fast* machining strategy available in Cerec MC XL does not degrade the marginal strength of the evaluated CAD/CAD materials when compared to its *fine-fast* machining strategy. Depending on the material, resin composites have the potential to perform better than some glass-ceramic materials.

## 1. Introduction

Computer-Aided Design/Computer-Aided Manufacturing (CAD/CAM) technology, from the 1980s onwards, has been established as a crucial part of the dental routine. The application of polycrystalline ceramics, for example, would have been inconceivable without the possibility of machine processing. Dentists benefit immensely from such technological breakthroughs, among many, in terms of materials, accuracy of fitting, and reduced treatment times. Nonetheless, a shadow is cast over this success story from clinical feedback. Exemplary, a retrospective *in vivo* study on 35 thousand CAD/CAM restorations showed that from all fractures within 3.5 years, 22.8% occurred during installation, mainly in crowns, and mostly from their cervical margins [1]. Fractographic analyses of zirconia frameworks fractured during service confirm the origin of failure at the margins, weakened by the processing of the green compacts before final sintering [2]. These are not isolated cases: recovered fractured crowns from private and public practices showed fracture initiation sites located predominantly at the cervical margins of CAD/CAM all-ceramic crowns [3,4].

Despite the undoubted efficiency of CAD/CAM technology, these and other clinical outcomes [5,6] reveal the irony: processing of dental materials by machining seems to contribute to the accelerated failure of dental restorations. Inevitably, cervical margins of crowns run thin – a fact that often can’t be prevented – it does come with the territory. Precisely on that edge, high hoop stresses concentrate during cementation, occlusal loading, and hygroscopic expansion of the cement. It is thus to expect that cervical margins behave as the weak link in crown-like geometries. Owing to their ductility, this circumstance is compensated in metallic alloys, though ceramics are more affected due to their high brittleness and low fracture toughness, compromising the chipping strength at the edges [7]. For feldspathic ceramics, for instance, strength losses up to 56% were calculated to result from machining with rotary diamond burs [8]. Even densely sintered zirconia was shown to lose about 40% of its strength due to mechanical processing. Defects in materials ground in a partially crystallized state lead to a dramatic loss of strength of up to 72%.

Still rare, *in vitro* test methods have recently attempted to simulate conditions where fractures originate from cervical margins [9], but standardization of such test set-ups is still lacking. Geometries and loading conditions still need to be converged among methodologies to render usable and mutually comparable mechanical parameters.

In this contribution, different classes of machinable restorative materials were processed by CAD/CAM using two machining strategies available in a popular commercial system. Using a newly developed hoop-strength test, model-crown sphero-cylindrical specimens were fractured and the inner marginal strength was evaluated using the finite element method with the objective to state on the effect of machining strategy on the strength degradation.

The tested hypotheses were: (i) *rough-fast* machining produces a higher density of marginal damage; (ii) *rough-fast* machining leads to lower inner marginal strength, and; (iii) ceramic-based materials show higher inner marginal strength when compared to hybrid materials.

## 2. Materials and methods

### 2.1. Materials and specimen preparation

The CAD/CAM materials used in this study are listed in Table 1 and encompass a wide range of different material classes and mechanical properties (i.e. Young’s modulus, flexural strength, and fracture toughness).

**Table 1.** Summary of CAD/CAM materials used, their descriptions of class, manufacturers, and lot number.

Material	Composition	Manufacturer	LOT	Young’s modulus (GPa)	Biaxial flexural strength (Weibull *σ*_0_) (MPa)	Weibull modulus *m*	*K*_Ic_ (MPa√m)
IPS e.max^®^ CAD	60.3 vol.% Li_2_Si_2_O_5_ 6.8 vol.% Li_3_PO_4_ 1.0 vol.% Cristobalite	Ivoclar-Vivadent, Liechtenstein	119044	102.5 [10]	647 [11]	17.4 [11]	2.04 [12]
Suprinity^®^ PC	27.1 vol.% Li_2_SiO_3_ 14.4 vol.% Li_2_Si_2_O_5_ 11.6 vol.% Li_3_PO_4_	Vita Zahnfabrik, Germany	66612	102.9 [10]	611 [11]	5.3 [11]	1.39 [12]
Enamic^®^	UDMA, TEGDMA (25 vol.%); Aluminosilicate glass (75 vol.%)	Vita Zahnfabrik, Germany	74770	37.4 [10]	195 [11]	19.3 [11]	1.25 [12]
Grandio^®^ Blocs	UDMA, BIS-EMA, TEGDMA (∼15vol.%); Glass fillers (∼85vol.%)	Voco, Germany	1817685	18 [13]	244 [14]	13.1 [14]	1.42 [14]
Lava™ Ultimate	Bis-GMA, Bis-EMA, TEGDMA, UDMA (∼25 vol.%); Silica and Zirconia fillers (∼75 vol.%)	3M Oral Care, Germany	899630	12.7 [10]	231 [14]	10.8 [14]	1.14 [12]

Bis-GMA: bisphenol A glycidylmethacrylate; Bis-EMA: ethoxylated bisphenol-A dimethacrylate; TEGDMA: triethylenglycol dimethacrylate; UDMA: urethane dimethacrylate; *K*_Ic_: fracture toughness.

**Figure 1.**
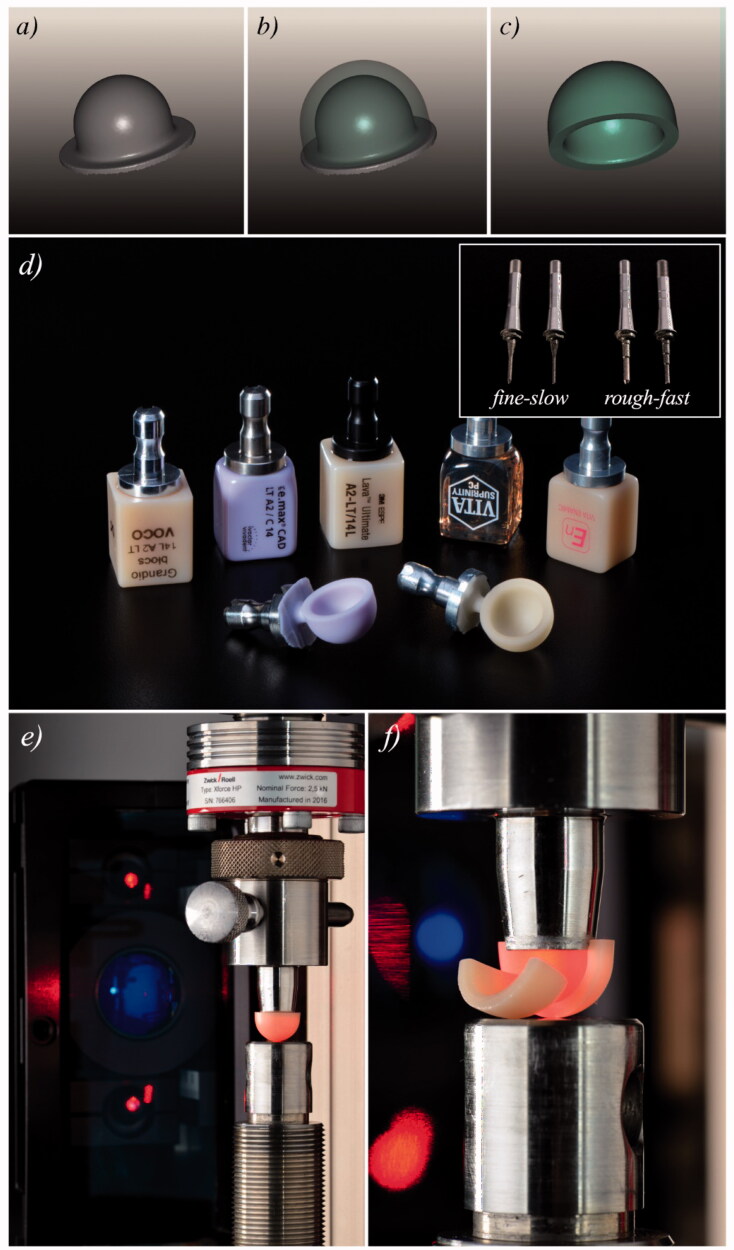
(a) Scanned abutment, (b,c) designed sphero-cylindrical shape to be machined, (d) CAD/CAM materials investigated in this study before and after machining (inset shows the burs used in the two different machining protocols), (e) marginal strength test is performed with a laser extensometer, and (f) a view of a fractured specimen [Reprinted with permission from Elsevier].

In the present study, the mechanical test method developed in Ref. [15] was employed to assess the inner marginal strength of the evaluated materials. To provide for the standardization of the geometrical shape to be machined, a model crown was designed using CAD software based on a metallic abutment with a specific form of a half-sphere with elongated cylindrical margins (Figure 1(a)) to render a sphero-cylindrical object (Figure 1(b,c)) as a crown-like specimen geometry. This shape differs from a dental crown by having a rotational symmetry around its long axis, a 0.5 mm internal margin radius, and a sharp external angle in 90° degrees. This was intended to allow a uniform loading contact around the internal axial wall just below the internal rounded margin and a homogeneous stress state over the inner margin’s circumference. The thickness of the specimens was set to 1.65 mm so to induce the highest marginal stress in the inner margin, the region where clinical marginal fractures are usually located [3,4]. Thinner margins tend to magnify the stress at the outer margins, where chippings at the 90° angle rim affect the failure mode [15].

The CAD/CAM blocks (see Figure 1(d)) were machined (*n* = 20) using a chairside CAD/CAM machine (Cerec MC XL, Dentsply-Sirona, USA) according to two machining protocols available in the system: *rough-fast vs. fine-slow*. The *fine-slow* option used one thin cylindrical pointed bur (12EF − 65 35 178) and one thin step bur (12EF − 65 35 186), as shown in the inset of Figure 1(d), and needed ∼30 min for each specimen. The *rough-fast* option used one thick cylindrical pointed bur (12S − 62 40 149) and one thick step bur (12S − 62 40 167) (see inset in Figure 1(d)), and took ∼15 min to machine one single specimen. The burs were renewed every five specimens. After machining, the peg connecting the specimen to the block was removed with an extra-fine diamond bur without touching the margin. The materials IPS e.max^®^ CAD and Suprinity^®^ PC were crystallized according to the manufacturers’ instructions in a calibrated Vacumat 4000 oven (Vita Zahnfabrik). No specimen was further polished before the mechanical test, to preserve the surface topography generated by machining.

### 2.2. Scanning electron microscopy

Two random samples of each material/machining protocol were washed in Isopropanol, dried, gold sputter-coated, and inserted in a Scanning Electron Microscopy (Leitz ISI SR50, Akashi, Japan). Three images were taken from different locations in the same sample and used to randomly select smaller and larger margin defects.

### 2.3. Hoop-strength test

Each set of specimens was dried in the oven (3 h at 110 °C for ceramic materials; 60 °C for composites) to eliminate surface humidity. The specimens were immediately immersed in hot silicon oil, in order to to protect the specimens against environmental water, which can induce slow crack growth in ceramic-based materials during the test.

The specimens were loaded with a metallic conical piston having lateral walls in 7° to simulate a naturally prepared tooth abutment (see Figure 1(e)), using a test termed ‘hoop-strength test’ developed in Ref. [15]. In this test, the walls of the loading conic piston touch the whole circumference on the inner wall of the sphero-cylindrical structures just below the rounded internal cervical margin. This creates uniform hoop stress along the entire circumference of the margin. The model crowns were loaded to fracture (Figure 1(e)) in silicon oil to prevent environmental crack growth and reduce the friction between the metallic piston and specimen. The force at fracture (in Newtons) was recorded for each specimen and used as input for the finite element simulation.

### 2.4. Finite element analysis (FEA)

A three-dimensional model was constructed based on the dimensions of the machined specimens (∼41,000 elements, ∼190,000 nodes) with the margin having a refined mesh, along with a model for the piston and the flat base made of isotropic steel (Young’s modulus = 200 GPa, Poisson’s ratio = 0.3). The loading set-up was simulated in a quarter model ensuring symmetry conditions using the physical properties of the corresponding restorative materials (see Table 1) to evaluate the stress distribution in the models (ANSYS R18.1). All materials were considered homogeneous and isotropic. The degrees of freedom at the base was set to zero, and the piston was constrained to the vertical movement. The friction coefficient between piston and model was set for all materials to µ = 0.17 [15]. The maximum fracture force obtained from each hoop-strength specimen was used to calculate the maximum principal tensile stress taking place at the inner margin of the sphero-cylindrical model, directly above the contact point between the piston and the specimen. This was the fracture location for all specimens as verified using fractography. The set of calculated stress values at the inner margin was evaluated using Weibull statistics and described by the shape (characteristic strength, *σ*_0_) and scale (Weibull modulus *m*) parameters according to DIN 843-5 [16]. Statistical difference was established as the failure to overlap the 90% bands of the confidence intervals.

To qualitatively validate the stress distribution in the model during the simulation, a real test set-up was performed using a highly translucent material (pre-crystallized Suprinity^®^ PC) while trans-illuminated by an automatic polarimeter having a spatial resolution of 11 µm/px (StrainMatic M4/120.33, ilis GmbH) [17]. A phase delay between the two decomposed waves from the original polarized light source is created in glass components under stress, which results in optical anisotropy (2 indices of refraction), induced here by the mechanical loading. The birefringence, or double refraction, is proportional to the induced stress, visualized as light retardation in nm using a processing software (StrainAnalyzer v.2015.190, ilis GmbH).

## 3. Results

**Figure 2.**
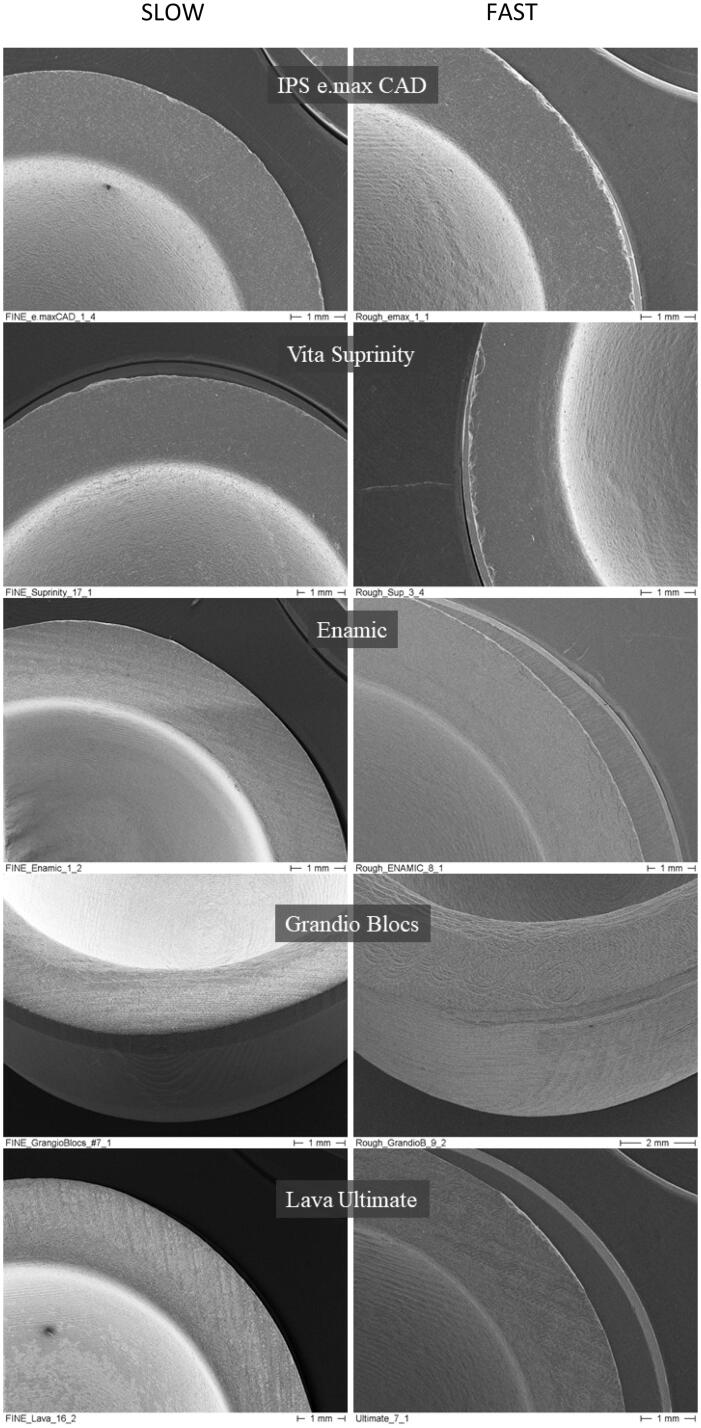
Scanning electron microscopy images of damaged surface on five different materials tested, in fine and rough protocol machining.

SEM analyses revealed damaged outer edges in the specimens of the five materials (Figure 2). However, it was possible to note that the *rough-fast* protocol produced more damage on the outer margin than did the *fine-slow* protocol for the ceramics when compared to composite materials. Suprinity^®^ PC and IPS e.max^®^ CAD had the most clearly defined chippings on the outer margin, whereas the resin composite materials (Enamic^®^, Grandio^®^ Blocs, and Lava™ Ultimate) showed almost no chippings. For the resin composites, no visual difference could be established in terms of the amount and severity of marginal damage between the machining protocols. Regarding the rounded inner margin, no differences can be stated on the surface quality based solely on the visual inspection under the SEM.

**Figure 3.**
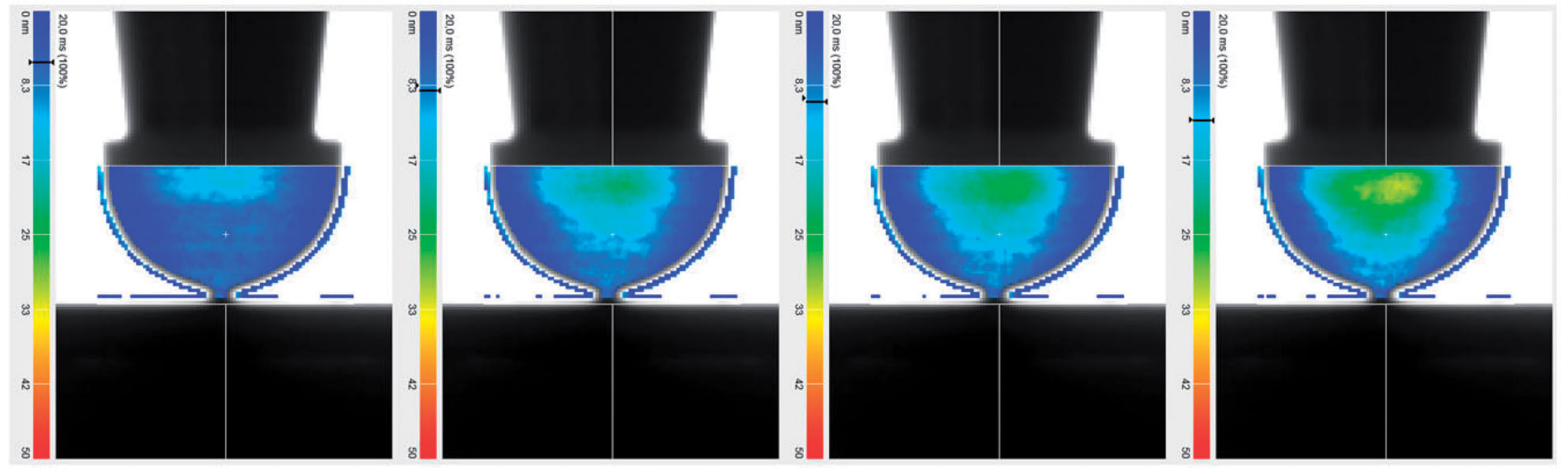
Light retardation illustrated using color scales from blue (0 nm, zero stress) to red (50 nm, high stress). The selected area for measurement is confined here to the region of the specimen in which there is light transmission (the upper region is excluded due to the opacity of the piston as it is inserted into the specimen lumen).

**Figure 4.**
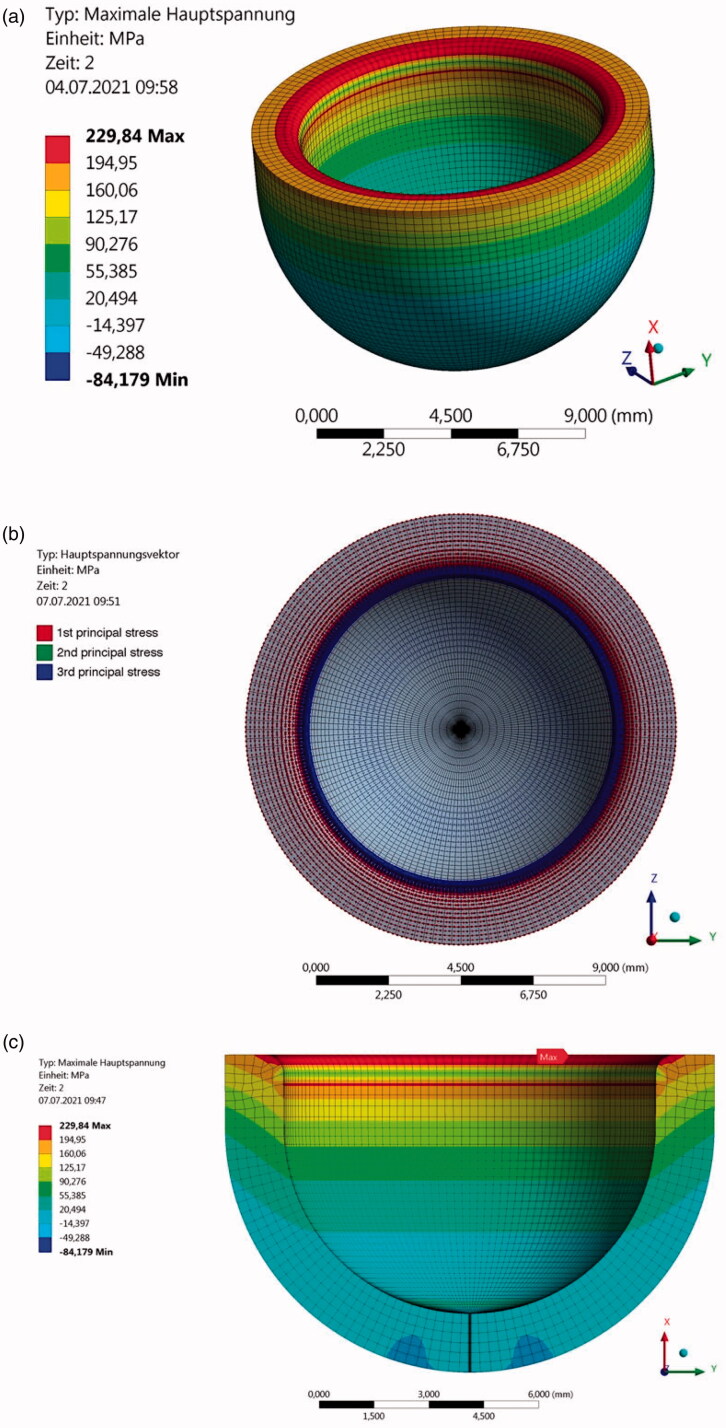
(a) FE Analysis of the maximum principal stress distribution in the sphero-cylindrical model crown for the material IPS e.max^®^ CAD; (b) principal stress trajectory plots presenting the circumferential stress directions; and (c) cross-section showing the maximum principal stress distribution through the thickness.

The stress distribution in the specimen, as seen using the polarimeter in time increments during loading in Figure 3, is distorted due to the convexity of the specimen and the increasing thickness of material towards the sides of the sphero-cylindrical geometry. The stressed area appears therefore confined to the center of the specimen and decreasing towards the sides; the only accurate distribution is at the centerline from the margin to the base. That stress profile distribution matched very well the one obtained by the FE simulation (see Figure 4).

The FE analysis confirmed that the contact point between the piston and the specimen generated circumferential compressive stress and a compensatory region of high tensile stress just above the contact point (see Figure 4). The highest tensile stress is located at the lower limit of the rounded section of the inner margin and decreases almost linearly toward the outer edge of the margin [15]. This trend of stress distribution was observed for all materials.

The Weibull parameters relative to the maximum principal stress at fracture are given in Table 2 for the two machining protocols. Of importance to note is the fact that for all materials no significant difference could be established between machining protocols, only among materials. The lithium disilicate IPS e.max^®^ CAD showed the statistically highest characteristic marginal strength, overperforming Suprinity^®^ PC. In the class of hybrid materials, Grandio^®^ Blocs performed superiorly than Lava™ Ultimate, Enamic^®^ and Suprinity^®^ PC . Enamic^®^ showed the lowest characteristic strength values among all materials.

**Table 2.** Weibull parameters were calculated for the inner marginal strength obtained from FE simulation of the obtained fracture forces in the *in-vitro* test.

Material	Rough-fast machining		Fine-slow machining	
	σ_0_ [90% C.I.]	*m* [90% C.I.]	σ_0_ [90% C.I.]	*m* [90% C.I.]
IPS e.max^®^ CAD	305.4 [287–324] aA	6.65 [4.9–9.1] aA	306.7 [282–333] aA	4.84 [3.6–6.6] bA
Vita Suprinity^®^ PC	199.9 [191–209] cA	8.90 [6.6–12.1] aA	214.6 [196–235] cA	4.21 [3.2–5.9] bB
Enamic^®^	124.2 [118–130] eA	8.53 [6.3–11.6] aA	118.4 [114–122] eA	11.6 [8.6–115.7] aA
Grandio^®^ Blocs	256.9 [248–266] bA	11.1 [8.2–15.0] aA	246.5 [236–257] bA	9.11 [6.9–12.4] abA
Lava™ Ultimate	154.3 [147–162] dA	8.31 [6.1–11.2] aA	147.1 [135–159] dA	4.93 [3.7–6.7] bA

Cells with the same uppercase letters in rows are not statistically different at *α* = 0.05.

Cells with the same lowercase letters in columns are not statistically different at *α* = 0.05.

## 4. Discussion

The marginal strength test employed here was designed to create a uniform hoop-stress in the inner margin of a simplified crown-like geometry using a cylindrical piston wedging the inner margin, a region implicated in clinical fractures of single crowns, whether monolithic or bilayered [3,4]. For that, all crowns were standard prepared in a dental CAD/CAM system having a homogeneous 1.65 mm thickness. Cervical crown margins are usually thinner than that, but as thinner the margin gets in this test configuration, the more the defects (chippings) on the outer margin begin to interact with the inner stress field and dominate the fracture mode, as shown in Ref. [15]. We aimed to avoid such failures, and induce inner marginal fractures. Also, thinner specimens could not be tested for composite materials due to their low elastic modulus, which caused the specimens not to fracture despite high vertical displacements of the piston into the specimen lumen.

Regarding inner marginal strength, no differences could be established between the *rough-fast* and the *fine-slow* machining protocols regardless of material class, despite the clear evidence of higher damage density induced by the *rough-fast* protocol to the outer margins of the ceramic materials. Finite Element Analysis gives insights in this regard, revealing that the highest tensile stress concentrated rather on the inner rounded margin without extending to the outer margin, which could trigger fractures from the observed chippings. Oilo et al. suggested this previously, namely, that the thinner the marginal wall is, the highest will be the tensile stress under hoop-stress states for the same applied load [4]. This has been corroborated in our previous study when calculating the stress at failure from critical defect sizes using fractography [15]. Lower volumes of material leading to higher stresses at fracture are also compatible with the expected Weibull behavior seen in most ceramic materials [18,19]. IPS e.max^®^ CAD, despite seemingly a Weibull material when natural defects are sampled in polished samples [11], has failed to conform to that expectation in this test configuration before [15]. That was due to the same type of defect having the same size distribution, reducing the critical stress for fracture of the thinner sphero-cylindrical specimens. The homogenization of the defect size was unexpectedly caused by the formation of a pore-rich zone on the machined surface, as a consequence of the partial melting of the ceramic smear layer. For such two-step ceramic materials that require a separate crystallization firing, the removal of this surface smear layer is therefore highly advised before further heat-treatment, not only on the outer surface but also on the intaglio of constructs.

A sufficiently adequate description of the observed mechanical behavior of the investigated materials can be easily squared within a mechanistic framework. A simplistic interpretation could seize in the ranking of the obtained marginal strengths to note the linear relationship with the fracture toughness (see Table 1), without risking any inappropriateness. The proportionality between strength and toughness has been previously reported, at least for dental ceramics [20]. Alternatively, taking the perspective of the critical defect size *a*_c_, using the available characteristic strength *σ*_0_ and fracture toughness *K*_Ic_, and solving the Irwin-Griffith equation for *a*_c_ (conducted here only for the *rough-fast* protocol, due to equivalence), namely, *a*_c_ = 1/π(*K*_Ic_/*Yσ*_0_)^2^, allows for more interesting insights. By generalizing a semi-elliptical crack geometry [21] produced by machining, characteristic critical crack sizes of 25.3 and 27.3 µm are obtained for IPS e.max^®^ CAD and Suprinity^®^ PC, respectively. Comparable *a*_c_ for both ceramics would be contradictory, once their respective *K*_Ic_ in the pre-crystallized state is 1.28 and 0.91 MPa√m [22]. However small, this difference would account for significantly longer cracks for Suprinity^®^ PC under supposedly equivalent surface stress during machining. The comparable critical crack sizes obtained here point therefore to one of two alternatives: in both materials, the defects are of the nature previously seen in the sphero-cylindrical IPS e.max^®^ CAD specimen in Ref. [15], that is, pores resulting from partial melting of the smear-layer; or homogenization in size occurred by consequence of the crack healing during crystallization, as described in Ref. [22] to occur in both materials. Against the latter plays the fact that the remaining crack is still too large, seen that in Ref. [22] cracks as large as 100 µm were healed to <5 µm during crystallization firing, a process driven by viscous flow of the glass and capillarity forces [23].

In hybrid materials, different mechanisms seem to be operational. For Enamic^®^, a characteristic critical crack size of 57.3 µm is obtained using the same approach. That order of magnitude fits well within the range of 20–60 µm, in which a second defect population within the parent population of Enamic^®^ has been shown to occur at a higher relative frequency for effective surfaces >0.43 mm^2^ and effective volumes >0.015 mm^3^ (compared to our test configuration herein) [24]. Those defects were depicted as polymer-infiltrated voids related to particle agglomerate shrinkage forming during partial sintering of the powder compact, having an elongated sharp geometry. It might therefore not be too far-fetched to expect the interplay of two defect types here too, with shorter machining cracks being responsible for the high-strength tail of the Weibull distribution, while the above-mentioned sintering voids being responsible for the lower tail of the distribution. For the two resin composites Grandio^®^ Blocs and Lava™ Ultimate, an *a*_c_ of 17.2 µm and an *a*_c_ of 31.2 µm are obtained, respectively, highlighting the role of filler packing (∼72 *vs.* ∼65 vol.%), filler shape and size [25], and the quality of the polymer-filler interface [26,27] in the toughening of dental resin composites. In all these aspects Grandio^®^ Blocs seem to benefit, reflecting a higher damage tolerance against machining.

Ultimately, based on the differences found in the SEM images of the machined samples, the hypothesis (i), that the *rough-fast* machining protocol would lead to a higher density of marginal damage was shown to be valid only for ceramic materials, if considered only the outer margin. The resin composites, however, showed no distinguishable differences regarding the outer marginal integrity when the *rough-fast* and *fine-slow* protocols were compared. This could play in favor of composite crowns to be employed with thinner margins since outer margin chippings tend to reduce the stress at failure for ceramic analogs. In terms of inner marginal strength, the lack of an effect extended also to ceramic materials, once no difference was found between the *rough-fast* and *fine-slow* protocol, negating hypothesis (ii). Due to the statistically higher characteristic marginal strength of Grandio^®^ Blocs compared to Suprinity^®^ PC, hypothesis (iii) could not be confirmed, exposing the potential for composites to compete with some ceramic materials for single crown applications.

It is important to note, nevertheless, that the differences in the machining protocols evaluated here are not related to the diamond grit size, which is the same in both sets of burs. It rather relates to the radii of the bur tips, which ultimately dictate the distance between parallel paths (resolution) taken during the machining strategy. Finer tips require shorter distances between passes, resulting in more time expenditure when compared to larger tips. The machining damage from the cutting by the diamond particles might therefore be comparable, as shown here using a mechanical test exposing the effect of surface damage on strength. The surface waviness, or microtopography, is obviously more affected by the machining protocol but shown here not to be meaningful when pertaining to marginal strength of ceramic and hybrid materials.

## 5. Conclusions

The two different machining strategies evaluated in this study did not affect the inner marginal strength of restorative materials, regardless of material type. Ceramic materials showed a higher density of chipping on the outer margin when compared to hybrid materials, which did not negatively affect the stress field of the inner margin.

Depending on the material, indirect resin composites seem to be able to compete with ceramic materials when it comes to the marginal strength of dental single crowns.
